# Mechanisms Underlying Hepatitis C Virus-Associated Hepatic Fibrosis

**DOI:** 10.3390/cells8101249

**Published:** 2019-10-14

**Authors:** Mousumi Khatun, Ratna B. Ray

**Affiliations:** Department of Pathology, Saint Louis University, 1100 South Grand Boulevard, St. Louis, MO 63104, USA; mousumi.khatun@health.slu.edu

**Keywords:** hepatitis C virus, hepatic stellate cells, fibrosis, exosomes, non-coding RNAs

## Abstract

Hepatitis C virus (HCV) infection often causes liver diseases, including fibrosis, cirrhosis and hepatocellular carcinoma (HCC). Liver fibrosis is the outcome of the wound healing response to tissue damage caused by chronic HCV infection. This process is characterized by the excessive accumulation of extracellular matrix (ECM) proteins, such as collagen fibers secreted by activated hepatic stellate cells (HSCs). Activation of HSCs from the quiescent stage is mediated by different mechanisms, including pro-inflammatory cytokines and chemokines released from HCV-infected hepatocytes and liver macrophages. HCV infection modulates the expression of different microRNAs that can be transported and delivered to the HSCs via exosomes released from infected cells, also leading to the development of advanced disease pathogenesis. Although recent advancements in direct-acting antiviral (DAA) treatment can efficiently control viremia, there are very few treatment strategies available that can be effective at preventing pathogenesis in advanced liver fibrosis or cirrhosis in patients. Assessment of fibrosis is considered to be the major part of proper patient care and decision making in clinical practice. In this review, we highlighted the current knowledge of molecular mechanisms responsible for the progression of liver fibrosis in chronically HCV-infected patients, and currently available methods for evaluation of fibrosis in patients. A detailed understanding of these aspects at the molecular level may contribute to the development of new therapies targeting HCV-related liver fibrosis.

## 1. Introduction

Hepatitis C virus (HCV) is a hepatotropic, positive-stranded RNA virus of the *Flaviviridae* family. HCV possesses 9.6 kilobases (kb) of single-stranded genome and encodes a polyprotein of around 3000 amino acids, which is cleaved by cellular and viral proteases. Based on the variations of genomic sequences, HCV is classified into seven genotypes and different subtypes, among which genotypes 1, 2 and 3 are more predominant than the others [1]. HCV infection is considered to be one of the major risk factors for liver-related pathogenesis. Approximately 85% of infected individuals develop chronic infection. The World Health Organization (WHO) suggests that more than 71 million people are chronically infected with HCV globally, and approximately 0.39 million infected people died due to HCV-related liver complications annually [2]. Chronic hepatitis C (CHC) infection leads to hepatic inflammation, which often stimulates liver fibrosis. Fibrosis is a consequence of wound-healing response, and is a continuous process of regeneration of damaged tissues by maintaining a balance between fibrogenesis and fibrolysis [3,4]. The inflammatory response leads quiescent hepatic stellate cells (HSCs) to activate and transdifferentiate into myofibroblasts. These myofibroblasts play a major role in fibrogenesis, as they are the main source of production of different components of extracellular matrix (ECM) to replace the damaged tissues [5,6,7]. Excessive deposition of ECM leads to scar formation that usually can be reversed by fibrolysis. Fibrosis is a dynamic process and may reverse upon resolution of HCV infection at an early stage [8]. However, chronic damage stimulating fibrogenesis and insufficient fibrolysis is associated with reduction of the reversibility potential even after the resolution of HCV infection. At this stage, fibrosis becomes more adverse and progresses towards liver cirrhosis (LC) [3,9]. A key determinant of fibrosis reversion in patients with HCV is associated with clearance of activated HSCs through apoptosis [7,10]. However, prolonged liver injury results in the increased thickening of septae due to enhanced cross-linking by tissue transglutaminases and resistance to proteolysis by metalloproteinases limiting the complete regression of fibrosis [7]. Most often HCV-associated hepatocellular carcinoma (HCC) is seen with a background of liver fibrosis or cirrhosis [11]. Although current treatment options such as direct-acting antivirals (DAAs) can efficiently remove viruses from human bodies in more than 95% cases, HCV elimination does not signify a liver disease cure, particularly in patients with advanced fibrosis or cirrhosis [12,13]. Thus, additional treatment may be needed for effective recovery from liver damage due to HCV-associated fibrosis in infected patients along with viremia.

## 2. Mechanisms of HCV-Associated Liver Fibrosis

### 2.1. Inflammation

Inflammation is a protective physiological process of the liver against most viral infections and tissue damage [14]. However persistent, uncontrolled inflammation is the hallmark of severe liver damage related to HCV infection, including fibrosis, cirrhosis and HCC [15,16]. Chronic HCV infection is positively associated with increased expression of different pro-inflammatory cytokines and chemokines from hepatocytes, liver residential macrophages (Kupffer cells) and different immune cells (macrophages, natural killer cells and dendritic cells) recruited to the liver [17,18]. Exposure to macrophages (including Kupffer cells) by circulating HCV induce inflammasome formation [17,19], although the mechanism is not fully understood. Inflammasome is a multiprotein cytoplasmic complex that senses viral pathogen-associated molecular patterns (PAMPs) through NOD-like receptors (NLRs), especially the NLR family pyrin domain containing 3 (NLRP3) [20]. IL-1β and IL-18 are two major cytokines produced by inflammasome-mediated pathways [21]. We and others have shown the upregulation of IL-1β and IL-18 expression from Kupffer cells exposed to HCV-infected hepatocytes in vitro [17,19].

The constant induction of pro-inflammatory cytokine production by hepatic macrophages in chronic hepatitis C (CHC) patients may help to recruit immune cells to the liver and induce the inflammatory response. This may lead to the activation of quiescent HSCs to secrete and deposit the ECM in the liver, resulting in the development of fibrosis and cirrhosis. An increased expression of transforming growth factor (TGF)-β1, a potent profibrogenic cytokine from HCV replicon cells, induces fibrogenesis-related gene expression for HSCs [22]. Interestingly, IL-1β does not involve activation of HSCs [23]. We demonstrated that primary human HSCs or LX2 (immortalized human hepatic stellate cells) cells with conditioned medium (CM) derived from HCV-exposed human macrophages enhanced the expression of both inflammatory markers, such as NLRP3, IL-1β, IL-6 and cysteine-cysteine chemokine ligand 5 (CCL5) and profibrogenic markers, such as TGF-β1, COL4A1, matrix metalloproteinase 2 (MMP2) and α-SMA. Further investigation revealed that CCL5 released from HCV-exposed macrophages/Kupffer cells was responsible for the induction of inflammatory and profibrogenic markers in primary human HSCs and LX2 cells [23]. NLRP3 also plays an important role in HSC activation and involves pro-inflammatory cytokine production, resulting in inflammation-induced liver fibrosis [24]. Together these studies demonstrate cross-talk among hepatocytes, liver macrophages and HSCs following HCV infection, for the development of fibrosis.

We have further shown HCV-induced cancer stem-like cells (CSCs) when implanted into immune-deficient mice activate stromal fibroblasts [25]. Several murine fibroblast activation markers are increased at the mRNA or protein level in a xenograft tumor model, suggesting the presence of tumor-associated fibroblasts (TAFs) and the migration of fibroblasts to form stroma. Subsequent studies have shown that HCV-infected hepatocytes express TGF-β and activate stromal fibroblast markers. Reactive oxygen species (ROS) are oxygen-containing free radicals that can induce DNA damage, oxidation of proteins and lipids. ROS-mediated liver injury plays an important role in cascade of liver fibrosis [26]. HCV induces TGF-β1 expression by stimulating ROS generation in infected Huh 7.5.1 cells, which in turn activate HSCs [27,28]. Together these studies strongly indicate that TGF-β from HCV-infected hepatocytes is one of the major players in activating HSCs towards fibrosis.

### 2.2. Role of microRNAs

MicroRNAs (miRs), approximately 20–22 non-coding RNAs nucleotides, are involved in many biological processes. miRs can induce messenger RNA (mRNA) degradation or translation suppression by binding with the complementary sequence present mostly in the 3′ untranslated region (UTR) of that mRNA [29]. Aberrant expression of different miRNAs has been noticed in different disease conditions, including HCV-induced liver fibrosis and HCC [30,31,32]. Circulating miRNA expression profiles may serve as a useful non-invasive marker for the diagnosis of advanced liver disease in HCV-infected patients. Differential expression of miRs has been reported in HCV-induced liver fibrosis and is involved in HSC activation [33,34]. Expression levels of let-7s, particularly 7a-5p, 7c-5p and 7d-5p, has been inversely correlated with severity of fibrosis stage in CHC patients. Downregulation of let-7 correlates with the activation of HSCs by targeting the TGF-β pathway in CHC patients [31,35]. miR-150 and miR-194 are also downregulated in primary HSCs isolated from bile duct ligated rats [36]. Overexpression of miR-150 and miR-194 in LX-2 cells results in a significant inhibition of proliferation without affecting apoptosis. Further, miR-27a and miR-27b can activate HSC proliferation by targeting retinoid X receptor α (RXRα), which is responsible for inhibition of the proliferation of HSCs. Downregulation of these two miRs in fully activated HSCs has resulted in restoration of the ability of quiescent HSCs, including increased accumulation of cytoplasmic lipid droplets and decreased HSC proliferation in vitro [37]. miR-29 level has been found to be significantly lower in HCV-infected patients, which has been linked to increased HSC activation and collagen secretion. Overexpression of miR-29 in LX2 cells results in proliferation reduction and suppression of collagen fiber expression [38]. The HCV-induced downregulation of miR-449a and miR-107 correlates with increased CCL2 (a major inflammatory chemokine) and STAT3 phosphorylation expression by targeting components of the interleukin-6 receptor (IL-6R) complex, such as IL-6R and JAK1, respectively, in infected patients resulting in hepatic fibrosis [39]. Downregulation of miR-449a also plays an important role in the modulation of YKL40 expression, a factor known to induce the synthesis of extracellular matrix and fibrosis through targeting the NOTCH signaling pathway components in HCV-infected patients [40].

Differentially expressed serum miRs are considered important non-invasive biomarkers for different stages of HCV-associated liver fibrosis progression in patients [41]. A study using an Egyptian patient cohort indicated that miR-16, miR-146a, miR-221 and miR-222 can be used as effective biomarkers for HCV-induced fibrosis, since these miRs are upregulated in early and late fibrosis, and miR-222 and miR-221 are important for their high sensitivity and specificity in late-stage fibrosis [42]. miR-122, the most abundant microRNA in the liver, was found to be negatively associated with fibrosis in HCV-infected patients [43,44]. Transfection of protease-competent or protease-mutants from the HCV NS3 genomic region into HSCs downregulates the expression of miR-122 in HSCs during fibrosis [44]. The protease-competent NS3 showed a greater effect of upregulation in the expression of different pro-fibrotic genes including α-SMA, COL1A1 and TIMP-1, along with the downregulation of miR-122. However, a significant correlation between miR-122 level and fibrosis in CHC patients infected with HCV genotype 1 was not observed [45]. Therefore, single-protein expression from HCV in the regulation of fibrosis needs to be carefully evaluated. miR-21 expression levels positively correlated with the fibrosis stages in CHC patients as well as a carbon tetrachloride (CCL_4_)-induced mouse fibrosis model [46]. miR-21 enhances TGF-β signaling by directly targeting its negative regulator, SMAD7, resulting in the induction of fibrogenesis. Another study demonstrated that HCV infection can induce expression of miR-16 level in both peripheral blood mononuclear cells (PBMCs) of the patients as well as in in vitro cell culture systems. The authors identified that miR-16 can bind to the 3′-UTR sequence of HGF and SMAD7 for the downregulation of these two factors in the presence of HCV [47]. Upregulated miR-200c in HCV- infected patients regulates the Src-kinase signaling pathway by directly targeting FAS-associated phosphatase 1 (FAP-1), a negative regulator of Src signaling, thus promoting hepatic fibrosis [48]. We observed a significant upregulation of miR-20a expression in the serum of HCV-infected liver fibrosis patients, with gradual increase from the early stage to the late stage of fibrosis, which may serve as a predictive biomarker of liver fibrosis detection in the early stage [30]. Collectively, these studies identified important roles played by microRNAs in HCV-induced liver fibrosis.

### 2.3. Long Non-Coding RNAs

Long non-coding RNAs (lncRNAs), a class of transcripts with more than 200 bp in length, are expressed in a cell-type specific manner [49,50,51,52]. Over the last few decades, lncRNAs have emerged as being associated with almost all cellular pathways, including cell proliferation, migration, apoptosis, cell-cycle regulation, invasion and in progression of different cancers in humans. Changes in expression level and localization of cells have been observed under different disease conditions, including with many cancers. Differential expression of a large number of lncRNAs were identified in viral infection, including HCV-infected patients in response to viral protein expression, antiviral response and virus-induced liver damage such as fibrosis, cirrhosis and HCC [53,54,55,56]. HCV may directly modulate the expression and function of lncRNAs to induce liver fibrosis. On the other hand, lncRNAs can directly target host factors such as miRNAs, to modulate different cellular pathways associated with initiation and progression of liver fibrosis in HCV infection. Unfortunately, the role of lncRNAs in HCV-induced liver fibrosis have not been well studied. The involvement of TGFβ-induced lncRNA-ATB in HCV-associated liver fibrosis is suggested by modulating the expression of miR-200a [57]. A significant upregulation of lncRNA-ATB and downregulation of miR-200a levels in liver tissues from patients with HCV-related fibrosis were observed. Knockdown of lncRNA-ATB significantly upregulates miR-200a expression and downregulates the expression of β-catenin. A different study showed that the same lncRNA targeted miR-425-5p to activate HSCs, and induced the production of collagen 1 in HCV-infected patients [58]. Thus, lncRNAs can be a good therapeutic target for HCV-associated liver fibrosis. Further studies will clarify the role of lncRNAs in the progression of advanced liver disease in HCV-infected patients.

### 2.4. Exosomes

**Figure 1:**
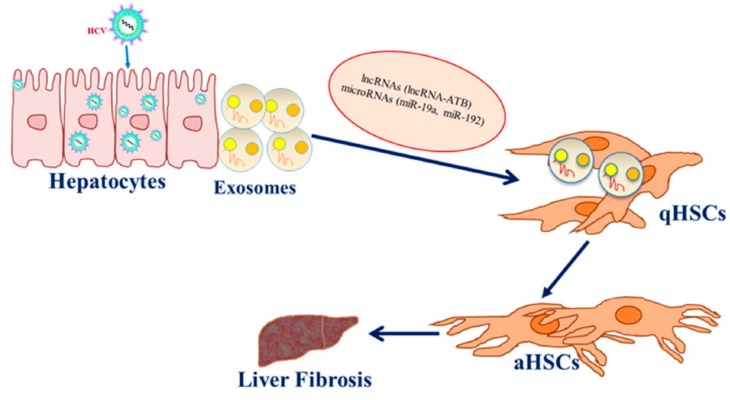
Exosomes carrying microRNAs and long non-coding RNAs (lncRNAs) released from hepatitis C virus (HCV)-infected hepatocytes enter into quiescent hepatic stellate cells (qHSCs) and activate hepatic stellate cells (aHSCs). This phenomenon triggers aHSCs towards liver fibrosis.

Exosomes are small extracellular vesicles (EVs) that are 30–100 nm in diameter, secreted by most of the cells via the exocytic pathway and are found in local and circulating body fluids [59,60]. Exosomes originate from the multivesicular endosome (MVE) and release from cells through fusion of MVEs to the plasma membrane [61]. Exosomes carry different biomolecules, including soluble and membrane-bound proteins, lipids, DNA, RNA, mRNAs, microRNAs and long non-coding RNAs, as cargo from one cell to another. Thus, exosomes serve as an important vehicle for intra- and intercellular communication without direct contact between different cells. Due to this feature, exosomes play an important role in many biological processes including immune responses and disease progressions, such as different cancer development and propagation of viral infections [60,61]. Different studies have indicated that exosomes are associated with HCV replication, spreading and antiviral response [62,63,64,65]. HC- induced miRs are transported from infected hepatocytes to HSCs by exosomes and activate HSCs leading to fibrosis (Figure 1). Transforming growth factor-β1 (TGF-β1), a major potent profibrogenic cytokine, plays an important role in the activation of HSCs and the production and accumulation of ECMs. We have shown that exosomes secreted from HCV-infected hepatocytes carry miR-19a, which is internalized into HSCs upon exposure and induces the expression of fibrogenic markers via the STAT3 mediated TGF-β signaling pathway [65]. HCV can also induce fibrosis by enhancing TGF-β1 expression and upregulating miR-192 expression via HCV core protein [66]. Upregulated miR-192 in HCV infection is transported to HSCs via exosomes to induce the activation of HSCs and the production of fibrogenic markers [67]. These studies indicate that exosomes are a good mediator for intercellular communication.

## 3. Fibrosis Markers

HCV-related liver fibrosis initiates a wide range of disease progressions in patients along with the alteration of normal architecture and function of the liver [68]. In an advanced stage of fibrosis, disruption of liver architecture due to septae and nodule formation causes increase in hepatic venous pressure called portal hypertension, which is a hallmark of liver cirrhosis (LC) [6]. Clinically, LC can be subdivided into (i) compensated liver disease, characterized by a portal pressure ≤ 10 mmHg, and (ii) decompensated liver diseases, defined by a portal pressure ≥ 10 mmHg, along with the risk of variceal bleeding, ascites, hyperbilirubinemia or encephalopathy of the liver [69]. More advanced stages lead to decompensation of the liver in the process of developing HCC. Therefore, detection of fibrosis at an early stage is the most important thing for potential reversal. There is an unmet need for assessment of the liver fibrosis stage during CHC infection for patient care and decision making by the clinicians. There are several semi-quantitative scoring systems such as METAVIR, Knodell and Ishak that are available for diagnosing liver fibrosis, and these are used in clinical practice [70]. METAVIR scores of 0, 1 and 2 have been defined as “no fibrosis”, “portal fibrosis without septa” and “portal fibrosis with few septa,” respectively. The clinical management of CHC patients depends on two advanced stages of liver fibrosis: (1) a METAVIR score of 3, defined by considerable fibrosis and histologically classified as septal fibrosis, and (2) a METAVIR score of 4, denoting cirrhosis, which necessitates specific and regular follow-ups for screening the status of HCC and esophageal varices [70].

Liver biopsy has been considered as the gold standard for fibrosis staging for many years, in order to histologically evaluate liver status [71]. However, this technique has drawbacks. For instance: (i) the histological evaluation depends upon a very small sample, often leading to underestimation of the exact situation of the liver; (ii) accuracy of the results from the biopsy sample depends upon the expertise of the pathologists; and (iii) it is an invasive as well as costly process that leads to limitations by applying to certain types of patients only.

Based on the limitations of liver biopsy techniques, several non-invasive and cost-effective detection methods are now considered in the evaluation of fibrosis stages. There are direct (class I) and indirect (class II) serum biomarkers for assessing fibrosis. Direct biomarkers include the metabolic products of ECM, including collagen fibers, hyaluronic acid (HA), YKL-40, laminin, fibronectin, metalloproteinases (MMPs), tissue inhibitors of matrix metalloproteinases (TIMPs) and transforming growth factor-β1 (TGF-β1), which have been produced during the remodeling of ECM via activated HSCs [5,72]. The indirect biomarkers consist of biochemical analysis of serum and the clinical parameters of patients, including the aspartate aminotransferase (AST)-alanine aminotransferase (ALT) ratio, the AST–platelet ratio index (APRI), FIB-4 (evaluation of fibrosis based on age, AST, ALT and platelet count), Fibro index (diagnosis based on platelet count, AST and gamma globulin) and Forns index (based on age, platelet count, cholesterol levels and Gamma-glutamyltransferase (GGT)) [73,74,75,76,77]. Egy-Score is a rising, non-invasive, serum panel composed of cancer antigen 19-9 (CA 19-9), age, α2-macroglobulin, total bilirubin, albumin and platelet count in a regression equation [78]. A retrospective study performed on 100 chronic hepatitis C naïve Egyptian patients showed that Egy-Score is a promising, accurate, easily calculated, cost-effective score in the prediction of hepatic fibrosis in chronic HCV patients with superiority over APRI, FIB-4 and Forns index in advanced hepatic fibrosis and cirrhosis [79].

Another useful non-invasive method for detecting fibrosis is transient hepatic elastography (TE), performed by FibroScan. In this technique, fibrosis stages are determined by measuring the liver stiffness using low-amplitude vibrations. Although it is a simple and efficient tool for detecting fibrosis, studies indicate that this technique should be used in association with other non-invasive biomarkers in HCV-infected patients [80,81].

Extracellular vesicles carrying specific genetic materials in HCV-infected patient serum can also be a strong diagnostic tool for analyzing fibrosis stages, as mentioned earlier. Serum pentraxin 3 (PTX3) level serves as a potential biomarker for the determination of significant (F ≥ 2) and advanced (F ≥ 3) fibrosis stages in HCV patients. PTX3 is an acute-phase protein rapidly produced by cells of innate immunity in response to inflammation. The levels of PTX3 are significantly higher in patients with advanced fibrosis compared to the F0–F1 stage, and significantly correlate with other indirect serum biomarkers as well as with the histological stage of liver fibrosis in HCV patients [82].

## 4. Therapies

Targeted therapies for liver fibrosis include strategies for the removal of injurious stimuli, inhibition of hepatic inflammation, suppression of HSC activation and profibrogenic mechanisms and induction of scar matrix degradation [3,9]. Recent understandings of fibrogenesis in regards to chronic liver disease have changed substantially. Previously, it was thought that the fibrosis process was irreversible, but current treatment strategies changed that assumption. Now fibrosis is considered to be a dynamic process with continuously ongoing fibrogenesis and fibrolysis, and sustained eradication of virus may result in a reduction of relevant extrahepatic disease manifestations [83,84].

The initial therapy for CHC was based on PEGylated (Peg) IFN-α and ribavirin (RBV). Although these treatments were less efficacious for elimination of HCV, improvement of fibrosis was observed in ~20% of infected patients [85]. The largest histological study, using the results of four clinical trials of CHC patients treated with IFN-α and ribavirin, revealed that out of the 153 patients with METAVIR F4 at baseline almost 50% reported an improvement of liver histology in their post-treatment liver biopsy, suggesting a regression of fibrosis [86]. However, due to the adverse side-effects of these treatments on patient quality of life, well-tolerated combinations of direct-acting antivirals (DAAs) have largely replaced interferon (IFN)-based therapy in later stages [87,88]. These treatments provide an extraordinary rate of sustained virological response (SVR, defined as HCV RNA negativity in a patient’s blood 6 months after completion of therapy) with over 95% cure rates reported in most trials involving all but type 3 HCV genotypes [12]. Fortunately for HCV genotype 3 infected patients, a second generation of DAAs with excellent SVR rates has already been introduced [89]. Patients who achieved SVR had increased survival benefits with fibrosis and a lower risk of hepatic decompensation and HCC occurrence [90,91,92,93]. Therefore, DAAs are recommended for use in all patients with significant liver fibrosis or cirrhosis, including decompensated cirrhosis [93]. Follow-up studies with HCV-infected patients up to 1 year after DAA treatment were found to have a minimal or zero decrease in HCC [94,95,96]. The occurrence of occult HCV infection (OCI) was detected in 15.0% of the DAA-based group and was more frequent in patients with genotype 3 [97]. OCI was significantly connected with severity of fibrosis and active inflammation post-SVR. The magnitude and frequency of fibrosis regression were lower in patients with OCI than in those without OCI. With that in mind, HCV-associated fibrosis is not solely dependent upon viral factors. Other factors, such as co-infection with different viruses, age and gender of the patients, duration of disease, obesity, alcohol intake and diabetes can also influence hepatic fibrosis. DAA may help in the reversal of fibrosis in the early stage; however, developing anti-fibrotic intervention is an unmet need in terms of curing patients.

## 5. Conclusions

**Figure 2:**
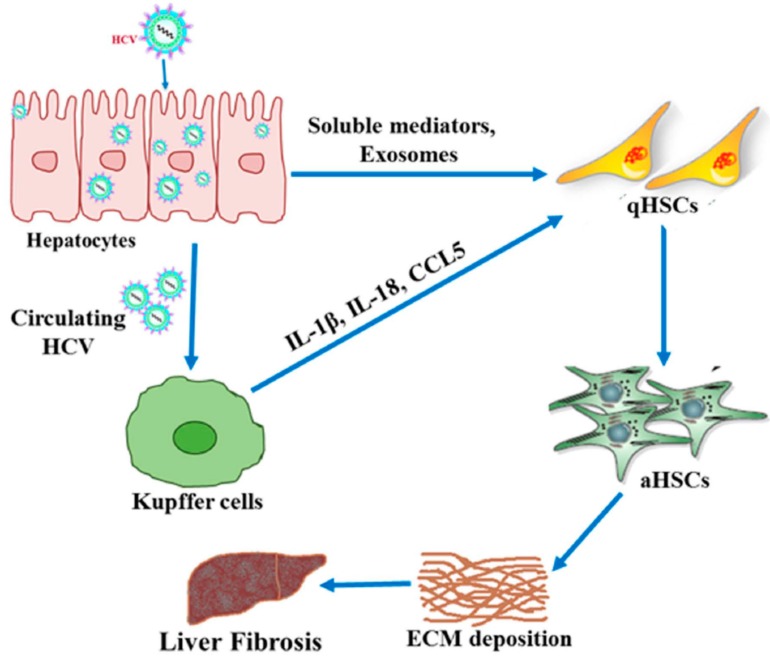
Communication among HCV-infected hepatocytes, quiescent HSCs (qHSCs) and Kupffer cells. HCV-infected hepatocytes secrete soluble mediators (cytokines/chemokines) and exosomes, and interact with qHSCs. On the other hand, circulating HCV released from infected hepatocytes interact with Kupffer cells and promote secretion of proinflammatory cytokines/chemokines, including IL-1β, IL-18 and CCL5. These mediators help quiescent hepatic stellate cells (qHSCs) activate HSCs (aHSCs), enhancing the deposition of the extracellular matrix (ECM) into the liver for fibrosis.

HCV infection is one of the major causes of hepatic failure due to advanced fibrosis, cirrhosis and HCC. HCV or exosomes released from the virus-infected hepatocytes interact with other liver cells and potentiate liver fibrosis (Figure 2). The mechanism of HCV-associated fibrosis is a multifaceted process, although HCV does not infect hepatic stellate cells [65]. HCV infection induces several factors in host cells, such as non-coding RNAs and cytokines/chemokines, which directly or through exosomes activate quiescent stellate cells. On the other hand, macrophages exposed to HCV induce soluble mediator like CCL5, which also stimulates HSCs for activation. Despite the development of DAAs with excellent SVR against HCV, not all the patients’ responded similarly to the regression of fibrosis. Positive outcome of the therapy mostly depends on early detection of the disease. Chronic hepatitis C infection is often asymptomatic, and early diagnosis and treatment of HCV reverses the fibrosis; however, HCV infection may result in end-stage liver damage due to the lack of early detection. Therefore, early detection and discovery of novel biomarkers for HCV infection-associated liver injury are very much needed. HCV infection alters the expression of different host genes, among which microRNAs can easily be used as potential biomarkers for the early detection of HCV-related liver fibrosis. Exosomes carry different macro and micro molecules from parent cells to the nearby HSCs to activate fibrogenesis. Further research on exosomes will provide a novel understanding of the initiation and progression of HCV-induced fibrosis, which may help to develop specific antifibrotic therapeutics. DAA treatment alone may not be sufficient for a complete cure of fibrosis, as several factors other than HCV are involved in maintaining liver disease pathogenesis. Since TGF-β is the most potent profibrogenic cytokine triggering activation and survival of HSCs, targeting these molecules may be an important approach to preventing fibrosis. However, targeting TGF-β may cause a serious outcome, as it is involved in several other essential cellular functions. Fibrosis is not evenly distributed in the liver, which can often lead to erroneous results in most conventional detection methods. The current uses of different non-invasive methods are appreciated by clinicians over invasive methods. However, some of the serum markers may be influenced by other physiological and metabolic statuses of patients, and thus should not be considered as the sole markers for HCV-related liver fibrosis. Therefore, both invasive and non-invasive methods are essential for assessing the proper degree of liver damage. One drawback of the system is not having a close animal model that HCV-associated fibrosis can mimic. Liver fibrosis is highly correlated with the activation of HSCs, and this is controlled by several factors such as cytokines/chemokines and microRNAs. Silencing these molecules using RNAi could be a promising strategy in reversing or controlling liver fibrosis. However, care should be given not to have an off-target effect. Thus, understanding host virus interactions in the development of liver damage, including efficient detection methods and developing anti-fibrotic modalities, may enhance the chances for a complete cure of patients suffering from HCV-associated liver fibrosis.
